# Non-Publication Is Common among Phase 1, Single-Center, Not Prospectively Registered, or Early Terminated Clinical Drug Trials

**DOI:** 10.1371/journal.pone.0167709

**Published:** 2016-12-14

**Authors:** Cornelis A. van den Bogert, Patrick C. Souverein, Cecile T. M. Brekelmans, Susan W. J. Janssen, Gerard H. Koëter, Hubert G. M. Leufkens, Lex M. Bouter

**Affiliations:** 1 Division of Pharmacoepidemiology and Clinical Pharmacology, Utrecht Institute for Pharmaceutical Sciences, Utrecht University, TB Utrecht, The Netherlands; 2 Central Committee on Research involving Human Subjects (CCMO), BH The Hague, the Netherlands; 3 National Institute for Public Health and the Environment (RIVM), Division of Public Health and Health Services, BA Bilthoven, The Netherlands; 4 VU University Medical Center, Department of Epidemiology and Biostatistics, MB Amsterdam, the Netherlands; Royal College of Surgeons in Ireland, IRELAND

## Abstract

The objective of this study was to investigate the occurrence and determinants of non-publication of clinical drug trials in the Netherlands.All clinical drug trials reviewed by the 28 Institutional Review Boards (IRBs) in the Netherlands in 2007 were followed-up from approval to publication. Candidate determinants were the sponsor, phase, applicant, centers, therapeutic effect expected, type of trial, approval status of the drug(s), drug type, participant category, oncology or other disease area, prospective registration, and early termination. The main outcome was publication as peer reviewed article. The percentage of trials that were published, crude and adjusted odds ratio (OR), and 95% confidence interval (CI) were used to quantify the associations between determinants and publication. In 2007, 622 clinical drug trials were reviewed by IRBs in the Netherlands. By the end of follow-up, 19 of these were rejected by the IRB, another 19 never started inclusion, and 10 were still running. Of the 574 trials remaining in the analysis, 334 (58%) were published as peer-reviewed article. The multivariable logistic regression model identified the following determinants with a robust, statistically significant association with publication: phase 2 (60% published; adjusted OR 2.6, 95% CI 1.1–5.9), phase 3 (73% published; adjusted OR 4.1, 95% CI 1.7–10.0), and trials not belonging to phase 1–4 (60% published; adjusted OR 3.2, 95% CI 1.5 to 6.5) compared to phase 1 trials (35% published); trials with a company or investigator as applicant (63% published) compared to trials with a Contract Research Organization (CRO) as applicant (50% published; adjusted OR 1.7; 95% CI 1.1–2.8); and multicenter trials also conducted in other EU countries (68% published; adjusted OR 2.2, 95% CI 1.1–4.4) or also outside the European Union (72% published; adjusted OR 2.0, 95% CI 1.0–4.0) compared to single-center trials (45% published). Trials that were not prospectively registered (48% published) had a lower likelihood of publication compared to prospectively registered trials (75% published; adjusted OR 0.5, 95% CI 0.3–0.8), as well as trials that were terminated early (33% published) compared to trials that were completed as planned (64% published; adjusted OR 0.2, 95% CI 0.1–0.3). The non-publication rate of clinical trials seems to have improved compared to previous inception cohorts, but is still far from optimal, in particular among phase 1, single-center, not prospectively registered, and early terminated trials.

## Introduction

Since decades, non-publication of trial results has been a major concern in clinical research, as non-publication causes research waste [1,2], and can bias evidence-based treatment guidelines and clinical decision making [3,4,5]. Research waste was defined by Chalmers and Glasziou as avoidable waste of investments in research due to inadequately producing and reporting, non-publication being one of its four stages [1]. In 2009, the magnitude of research waste in clinical research was estimated at 85% [1]. Moreover, non-publication is unethical because the burdens and risks imposed on study participants do not contribute to the body of knowledge.

The waste and bias implicated in clinical research caused by non-publication over the past years [3,6,7,8,9,10,11,12,13,14,15,16,17,18] has strengthened the view of several organizations and governments that all clinical trials must be published [19,20,21,22,23]. Previous studies specifically focused on publication of randomized controlled trials (RCTs) [24], covered only trials within one medical specialty [25], examined a limited selection of determinants, or used incomplete trial cohorts depending on public registrations [26,27] or interview response rates [10]. The most well-known determinant for non-publication is having a ‘negative’ outcome [28], but other reasons for non-publication have been proposed as well [29]. Thus, there is limited data on the occurrence of non-publication and its determinants that is both recent and complete. Investigating determinants of non-publication can identify and provide specific solutions for areas where the problem of research waste and bias is most persistent. Therefore, the aim of our study was to investigate the occurrence and determinants of non-publication of clinical drug trials in a country-wide inception cohort of clinical drug trials.

## Methods and Data Collection

The design of our study and the characteristics of the included trials have been published elsewhere [30]. In short, the inception cohort consisted of all clinical drug trials reviewed by IRBs in the Netherlands between 1 January and 31 December 2007. We used ToetsingOnline [31], the database of the competent authority of the Netherlands (the Central Committee on Research Involving Human Subjects, abbreviated in Dutch as CCMO), the only source containing a complete record of all trials that underwent IRB-review, to identify the cohort, the determinants, and the stages of progress of the included trials. In addition, we searched the trial registries clinicaltrials.gov and ISRCTN for the candidate determinant prospective registration, and for the availability of trial results in public registries. We originally defined prospective registration as registration before the first patient is recruited [30]. Because start-of-trial dates were missing in the database, we changed the definition of prospective registration to registration within one month of IRB-approval. In our experience, most trials start recruitment later than one month after IRB-approval, so this threshold classified more not prospectively registered trials as prospectively registered than vice versa. Sensitivity analyses were performed using two less strict thresholds of prospective registration: registration within 1 year of IRB-approval, and registration at any moment.

The search algorithm for publications used the platforms Pubmed, Embase and Google Scholar. More details are reported in the protocol [30]. We conducted the final search for publication and availability of results in January and February 2016. So, the follow-up since IRB-approval was 8 years at minimum, and 9 at maximum. Questionnaires were e-mailed to the principal investigators (PIs) of the trials, asking for reasons for non-publication. If the PI had left the company or the hospital that conducted the trial, we tried to contact the PI at his current affiliation, or otherwise we attempted to contact colleagues of the PI that were involved in the same trial. After identification of the right person, at maximum two reminders were sent. The Dutch accredited IRBs were asked for permission to send the questionnaire to the PIs. All IRBs consented and provided a signed letter of endorsement, which we attached to the questionnaire. The list of 23 Dutch accredited IRBs can be found on the website of the CCMO [32].

Candidate determinants were trial characteristics that the PI filled out on a form at the time of submission of the trial application for IRB-review. This form is mandatory and identical for all IRBs in the Netherlands. Prospective registration on the registries of clinicaltrials.gov or ISRCTN, and whether the trial was completed as planned or terminated early were also candidate determinants.

To be consistent with the literature referred to above, and for the purpose of linguistic clarity, we used publication as an outcome rather than non-publication. A publication was defined as a peer-reviewed article (i.e. the reciprocal of non-publication). Percentages of published trials were calculated for each of the determinant categories. Logistic regression was used to calculate crude and adjusted odds ratios (ORs) and 95% CIs for the association between determinants and publication. The final multivariable model included determinants that were retained after backward stepwise elimination based on the likelihood ratio, using p>0.2 as elimination rule. The original published study protocol prescribed Cox-regression for multivariable analysis instead of logistic regression [30]. However, the hazard ratios of determinants were not proportional during the observation period. Moreover, the end-of-trial dates were missing for 186 trials. Therefore, the date of IRB-approval was used as the starting point of follow-up, instead of the end-of-trial date prescribed by the protocol [30]. Because we were unable to control for the duration of the trials, interpretation of the hazard ratio would therefore be challenging and we decided to use logistic regression instead. The Kaplan Meier analysis was used to visualize the cohort from its starting point (date of IRB-review) until the endpoint (publication or non-publication), stratified by trial phase, one of the determinants which also discriminates between longer- and shorter-during trials [33].

We also stratified by oncology versus other disease areas (pre-specified in the protocol), and further stratified oncology trials by phase 1 trials versus other phase trials (post-hoc). Oncology phase 1 trials differ from other disease area phase 1 trials in that oncology phase 1 trials are usually restricted to patients, while most other disease areas include healthy volunteers [34].

In a second post hoc analysis, we investigated the association of the direction of results and publication. We categorized the direction of conclusions as positive, negative or descriptive. This categorization was based on the conclusion paragraph of the publication (e.g. the investigated treatment was superior, equivalent, and/or safer than the comparator), and for the unpublished trials on the primary outcome measurement reported in the registry (positive if the primary outcome was in favor of the investigated treatment, negative if not, and descriptive if no statistical test was provided in the registry). All data analyses were performed in IBM SPSS Statistics, version 23.

## Results

**Fig 1:**
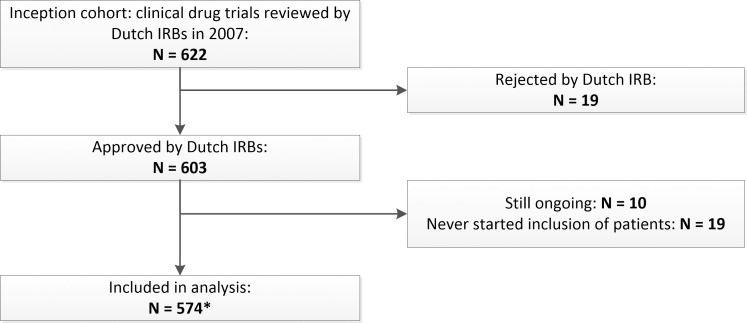
Stages of progress of the inception cohort. IRB = institutional review board. The end-of-trial form was missing of 186 of the 574 (32%) trials that were included in the analysis. Principal investigators of 73 of these trials responded to our questionnaire, completing the information on the end-of-trial. From the remaining 113, of 87 trials we found other documents than the end-of-trial form indicating that the trial had started (for example, emails from the IRB or amendments), or we found that the trial was published.

Of the 622 trials reviewed by the Dutch IRBs, 19 (3.0%) were rejected, and after obtaining IRB-approval, another 19 trials never started the inclusion of patients (Fig 1). Thus, before any patients were included, 6% of the trials had reached their final stage of progress. Of the 574 trials that started, 334 trials (58.2%) were published within the observation period of 8–9 years after IRB-approval.

Of 26 trials included in the analysis we had no follow-up information. The 113 trials with missing information about completion were assumed to be completed as planned.

Table 1 shows all candidate determinants and the percentages of publication for each determinant category. Nine of these candidate determinants were included in the multivariable logistic regression model (Table 2). In this model, phase 2 (adjusted OR 2.6; 95% CI 1.1–5.9), 3 (adjusted OR 4.1; 95% CI 1.7–10.0) and other-phase trials (adjusted OR 3.2; 95% CI 1.5–6.5) had a significantly higher likelihood of publication compared to phase 1 trials. Trials of which the investigator or company was the applicant had a significantly higher likelihood of publication compared to trials of which a contract research organization was the applicant (adjusted OR 1.7; 95% CI 1.1–2.8). Furthermore, international multicenter trials within the EU (adjusted OR 2.2; 95% CI 1.1–4.4) or also outside the EU (adjusted OR 2.0; 95% CI 1.0–4.0) were more likely published than single center trials. Invasive observational trials had a lower likelihood of publication compared to intervention trials (adjusted OR 0.4; 95% CI 0.2–0.9). Trials that were not prospectively registered had a lower likelihood of publication compared to prospectively registered trials (adjusted OR 0.5; 95% CI 0.3–0.8). Sensitivity analyses showed that the magnitude of this association increased if the threshold of prospective registration was changed to registration within one year of IRB-approval, or to registration at any moment (data not shown). Finally, trials that were terminated early had a substantially lower likelihood of publication compared to trials that were completed as planned (adjusted OR 0.2; 95% CI 0.1–0.3).

**Table 1:** Frequencies and publication percentages of candidate determinants.

	N in analysis (% published)
**All trials included in the analysis**	574 (58.2%)
**Sponsor**	
Pharmaceutical industry	352 (59.1%)
Investigator (industry (co-)funded)	71 (52.1%)
Investigator (no industry funding involved)	151 (58.9%)
**Phase**	
Phase 1	119 (34.5%)
Phase 2	130 (60.0%)
Phase 3	172 (72.7%)
Phase 4	57 (56.1%)
Other than phase 1–4*	96 (60.4%)
**Applicant**	
Contract research organization	214 (50.0%)
Investigator or company	360 (63.1%)
**Centers**	
Single center	249 (45.4%)
Multi center only in the Netherlands	54 (53.7%)
Multi center in the Netherlands and the EU	82 (68.3%)
Multi center in the Netherlands and outside the EU	189 (72.0%)
**Therapeutic effect expected**	
Therapeutic effect expected^†^	356 (64.6%)
No therapeutic effect expected	218 (47.7%)
**Type of trial**	
Intervention	517 (59.8%)
Invasive observational^‡^	45 (42.2%)
Non-invasive observational	12 (50.0%)
**Approval status of drug(s) in trial**	
Unapproved drug(s) in trial	306 (54.6%)
All drugs in trial approved, studied outside approved indication	147 (65.3%)
All drugs in trial approved and studied within approved indication	121 (58.7%)
**Drug type**	
Regular medicinal product	549 (57.7%)
Special drug category involved^§^	25 (68.0%)
**Participant category**	
≥18 years old and mentally capacitated	532 (58.6%)
<18 years old and/or mentally incapacitated	42 (52.4%)
**Disease area**	
Oncology	113 (66.4%)
Other disease areas	461 (56.2%)
**Prospective registration**^**||**^	
Prospectively registered	215 (74.9%)
Not (prospectively) registered	359 (48.2%)
**Completion**	
Completed as planned	472 (63.6%)
Terminated early	102 (33.3%)

*Studies not primarily intended to provide information about the drug, nor conducted within the context of a drug development program.

^†^ Trials were regarded as therapeutic if it is reasonable to assume that participation will be of direct clinical benefit to the subject.

^‡^ In observational trials, the investigator does not seek to change the observed situation, but simply to describe and record it as accurately as possible. Invasive procedures concern the penetration of the skin or mucosa with the aid of instruments, X-rays or magnetic resonance, or the introduction of an instrument into the body, or psychologically invasive observational research, involving the experimental creation of an unaccustomed situation which may give rise to negative emotions in the subject.

^§^ Vaccine, radiopharmaceutical, somatic cell therapy, antisense oligonucleotide.

^||^ Prospective registration was defined as registration of the trial at www.clinicaltrials.gov or www.isrctn.com, at latest one month after IRB-approval.

**Table 2:** Associations between determinants and publication, expressed as crude and adjusted odds ratios (OR), and 95% confidence intervals (CI) of the crude and adjusted ORs.

Determinants		Crude OR (95% CI)	Adjusted OR (95% CI)
**Phase**	** **		
** **	Phase 1	ref	ref
** **	Phase 2	2.9 (1.7–4.8)	2.6 (1.1–5.9)
** **	Phase 3	5.1 (3.1–8.4)	4.1 (1.7–10.0)
** **	Phase 4	2.4 (1.3–4.6)	2.4 (0.9–6.3)
** **	Other than phase 1–4	2.9 (1.7–5.1)	3.2 (1.5–6.5)
**Applicant**			
** **	Contract research organization	ref	ref
** **	Investigator or company	1.7 (1.2–2.4)	1.7 (1.1–2.8)
**Centers**			
** **	Single center	ref	ref
** **	Multicenter only in the Netherlands	1.4 (0.8–2.5)	1.2 (0.6–2.4)
** **	Multicenter in the Netherlands and the EU	2.6 (1.5–4.4)	2.2 (1.1–4.4)
** **	Multicenter in Netherlands and outside EU	3.1 (2.1–4.6)	2.0 (1.0–4.0)
**Therapeutic effect expected**			
** **	Therapeutic effect expected	ref	ref
** **	No therapeutic effect expected	0.5 (0.4–0.7)	1.7 (0.9–3.3)
**Type of trial**			
** **	Intervention	ref	ref
** **	Invasive observational	1.5 (0.5–4.7)	0.4 (0.2–0.9)
** **	Non-invasive observational	0.7 (0.2–2.6)	0.9 (0.3–3.2)
**Participant category**			
** **	≥18 years old and able to provide consent	ref	ref
** **	<18 years old and/or unable to provide consent	0.8 (0.4–1.5)	0.5 (0.2–1.0)
**Disease area**			
** **	Oncology	ref	ref
** **	Other disease areas	0.7 (0.4–1.0)	0.7 (0.4–1.1)
**Prospective registration**			
** **	Prospectively registered	ref	ref
** **	Not (prospectively) registered	0.3 (0.2–0.5)	0.5 (0.3–0.8)
**Completion**			
	Completed as planned	Ref	ref
	Terminated early	0.3 (0.2–0.5)	0.2 (0.1–0.3)

**Fig 2:**
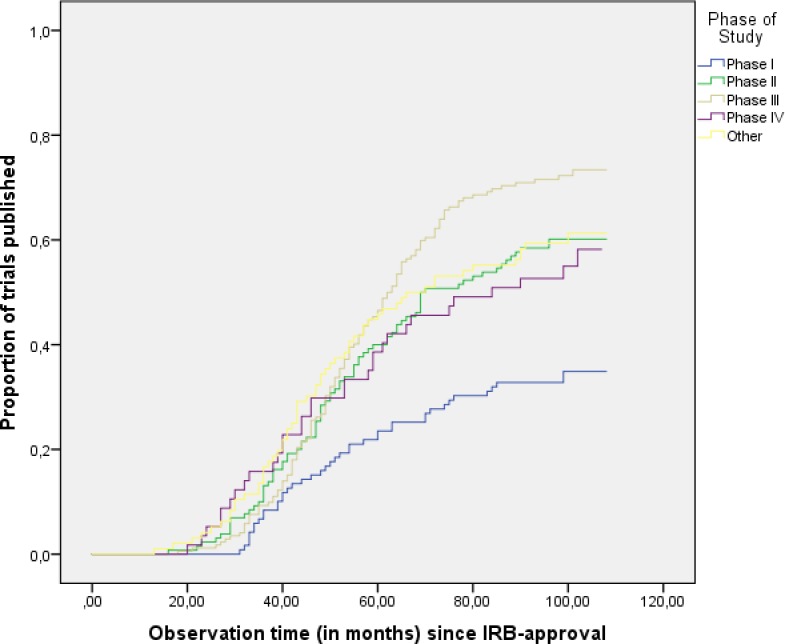
Kaplan Meier analysis of the publication rates of trial phases.

Based on visual inspection of the Kaplan Meier analysis, the curves of all phases seemed to approach their plateau after 8–9 years of follow-up since IRB-approval (Fig 2). The overall median time to publication since IRB-approval was 53 months (interquartile range (IQR) 39–65) and was not different between the trial phases.

Overall, non-oncology trials had a lower likelihood of publication compared to oncology trials; however, this association was not significant in the multivariable analysis (Table 2, adjusted OR 0.7, 95% CI 0.4–1.1, S1 Fig). No significant difference was observed in the median time to publication between other disease area and oncology trials (median time to publication 52 months (IQR 41–69) vs. 57 months (IQR 39–63), respectively). Post-hoc analysis showed that only 28 out of 100 (28%) other disease area phase 1 trials were published, which was significantly lower compared to the 13 out of 19 (68%) published oncology phase 1 trials (OR 0.2, 95% CI 0.1–0.5; S2 Fig). Among other phases we observed no difference in publication of other disease area and oncology trials (64% vs. 66%, respectively; S3 Fig).

Substantially more published trials (113/334, 34%) had also uploaded a summary of results in the clinicaltrial.gov or ISRCTN registries compared to the unpublished trials (23/240, 10%). Post hoc analyses showed that of the published trials, 42% of the direction of conclusions was positive, 19% was negative, and 39% were descriptive. Of the unpublished trials that reported results in their registry, 5 (22%) trials reported a positive primary outcome, 2 (9%) reported a negative primary outcome and 16 (70%) were descriptive or missing (primarily due to missing statistical information that was needed to infer a direction of the results).

The principal investigators of only 55 of the 240 (23%) unpublished trials responded to the questionnaire and provided the reason(s) for non-publication (S1 Table). The most important reason for non-publication among the responders was that the investigators had other priorities than to write a manuscript (18.2%). Other reasons included no statistically significant or clinically relevant results (14.5%), the manuscript was rejected by a journal (12.7%), the article was not finished yet (10.9%), and the study was underpowered due to poor inclusion of participants (10.9%).

## Discussion

Of the clinical drug trials approved by the Dutch IRBs in 2007, 42% had not been published as a peer-reviewed article by January/February 2016. The publication rate approximated their plateau at the time of our final search, suggesting that only a few more publications can be expected. The observed publication rate of 42% is relatively high compared with other studies investigating older cohorts [3,6,7,8,9,11,12,13,14,16,35,36]. This suggests that the publication rate of clinical trials has somewhat improved, but is still far from ideal. In particular, the publication percentage of the phase 3 trials (mainly RCTs) in our cohort (73%) was higher compared to previous cohorts investigating RCTs (overall, 54% published) [16,37]. Other recent research also supports that publication of phase 3 trials has been improved [17]. So, the regularly mentioned number of 50% non-publication [38] probably needs to be updated with regard to the phase 3 trials. Awareness-raising public campaigns [39], incorporation of publication requirements in clinical trial legislation [40] and advocacy by influential organizations [20] over the past decade may have contributed to this improvement. However, it is uncertain whether the identified publications have adequately reported all relevant aspects of the trials [41]. We are investigating this in the next phase of our cohort study [30].

The implicated research waste is considerable. Starting with the inception cohort of 622 IRB-reviewed trials, at least 140 (23%) failed to be completed as planned (Fig 1, Table 1). If we consider the published trials and the trials that are still running as not (yet) wasted, waste is implicated in 50% of the trials. This percentage should not be compared to the research waste estimate of 85% (of which 50% was due to non-publication) suggested by Chalmers and Glasziou [1], as we did not factor in research waste due to a poor design, conduct, data analysis, and selective reporting within the publications. Some waste is probably unavoidable (for example, trials sometimes are terminated early for ethical reasons). However, the need for better solutions is urgent considered the large public and private investments involved in the unpublished trials. Furthermore, 42% non-publication implies that publication bias in clinical drug trials is likely still substantial, despite many years of attention to this topic [42].

A limitation of our study was that we did not include the direction, magnitude and statistical significance of the trial results as determinants in our analysis. Previous studies included this determinant [10,15], by interviewing the PIs [10], or using trial reports submitted to the IRB [15]. However, this approach excludes trials of which no such data is available, potentially introducing selection bias. This would have excluded 113 of the 240 (77%) unpublished trials from our cohort. Furthermore, it is questionable how objective investigators can judge the direction of results of their own research [43], and definitions of ‘positive’ and ‘negative’ results are heterogeneous [28]. Despite the attached endorsement letters from the local IRBs, the response rate to our questionnaire was low. Among the responders, only 14.5% of the PIs reported that lack of significance or relevance of the results were a reason for non-publication. Having other priorities was the most common reason. Rejection by a journal was also among the most common reasons for non-publication. Both these reasons have been reported previously in the literature [16,44]. The post hoc analysis of the results of the unpublished trials that were uploaded in their registry demonstrated that these results sections are often incomplete and provide therefore little information on the influence of the direction of the results on the likelihood of publication. Furthermore, this finding suggests in line with other studies that uploading results in trial registries should be done more often, and that the quality of these results uploads needs improvement [45,46].

The publication rate of phase 1 trials was substantially lower compared to other phases. This has been shown before [8]. However, the percentage of phase 1 trials that was published in our cohort was substantially higher (35%) than the previous study (17%) [8], suggesting that progress has also been made in the field of phase 1 trials, but still not sufficient. Publication of phase 1 trials may be considered less interesting because their direct impact for clinical practice is limited when the drug is still far from marketing approval. Yet, phase 1 trials are an important source for the clinical pharmacology of drugs. Furthermore, data from previous phase 1 trials on similar drugs is essential in determining the risk of phase 1 (first in man) trials upfront [47]. Increasing transparency in general in this field of clinical research should be high on the agenda of regulators and the pharmaceutical industry, as emphasized by the slow release of information after the recent tragic events in a phase 1 trial in France [48].

Our post hoc finding that oncology phase 1 trials are more likely to be published than phase 1 trials in other disease areas suggests that inclusion of patients who are typically very ill [49] may positively influence publication of phase 1 trials. Or, argued differently, oncology phase 1 trials are in fact phase 2 trials, as phase 2 trials in most other disease areas are usually the ‘first-in-patient’ trials. The publication percentage of oncology phase 1 trials in our cohort was indeed similar to that of the phase 2 trials (68% and 60%, respectively).

The lower likelihood of publication of single center trials compared to multicenter trials has been shown in previous research [10]. In our cohort, this trend was visible, but only statistically significant for multicenter trials conducted also outside the Netherlands. Opportunities for increasing the incentive to publish exist at the level of the trial center. Publication metrics (including, but not limited to the number of trials published divided by the total number of trials conducted) should be reported on the center-website as well as the website of the local IRB for all trials conducted in the center [50]. Transparency about the local publication practices may stimulate stakeholders to require publication of all trials.

Invasive observational trials had a lower likelihood to be published compared to intervention trials. This association was not observed between observational non-invasive trials and intervention trials. Findings by other studies regarding this determinant are inconsistent [51] and the poor precision makes this determinant difficult to interpret.

We found that prospective registration in a trial registry was associated with publication. The idea of prospective registration of all trials was proposed many years ago [4], but in our cohort, only 37% of the trials were prospectively registered. The sensitivity analyses showed that the significant association with publication remained when using the less strict definition of prospective as registration within 1 year of IRB-approval. Since 2007, prospective registration has become increasingly mandatory, and higher registration rates have been reported [52]. But given the changes in the requirements for prospective registration since the inception of this cohort, higher publication rates cannot be predicted from this rise in prospective registration. Furthermore, there is no evidence that registries in their current state can adequately replace journal articles as the primary source for clinical guidelines, decision making and designing future trials. Until the issues with registries, such as completeness and quality of uploads of trial results, are solved, the peer-reviewed journal article remains the golden standard for reporting the results of clinical trials, and all clinical trials should be published as such.

### Conclusion

Our study shows a non-publication rate of clinical trials of 42%, which seems to be an improvement compared to previous inception cohorts, but is still far from optimal. Determinants of non-publication are early termination, no prospective registration, phase 1, and single center. Considerable research waste is implicated, and the likelihood of publication bias is high.

## Supporting Information

S1 Table Reasons for non-publication as reported by the responding principal investigators (PIs) to our questionnaire. In total, PIs of 55 out of 240 non-published trials responded. PIs could provide more than 1 reason.
(DOCX)

S1 Fig Publication rate of all trials stratified by oncology versus non-oncology (TIF)

S2 Fig Publication rate of phase 1 trials stratified by oncology versus non-oncology (TIF)

S3 Fig Publication rate of non-phase1-trials stratified by oncology versus non-oncology (TIF)

S1 File Anonymized dataset used for the analyses (XLSX)

S2 File Codebook of the dataset (PDF)

S3 File Questionnaires. Based on our initial search, we sent 4 different questionnaires, depending on whether or not we found that the trial was published, and depending on whether or not we had information on the end of trial (completed as planned or terminated early).
(ZIP)
