# Supplementary material for: Non-Publication Is Common among Phase 1, Single-Center, Not Prospectively Registered, or Early Terminated Clinical Drug Trials
Source: PLoS One. 2016 Dec 14;11(12):e0167709. doi: 10.1371/journal.pone.0167709 (PMC5156378; doi:10.1371/journal.pone.0167709)
Supplement: S1 Table — In total, PIs of 55 out of 240 non-published trials responded. PIs could provide more than 1 reason. (DOCX) [file pone.0167709.s001.docx]

| **Reason for non-publication** | **N** | **Percentage of the 55 responding PIs** |
| --- | --- | --- |
| Other priorities | 10 | 18.2% |
| Results not clinically relevant | 7 | 12.7% |
| Manuscript rejected by journal | 7 | 12.7% |
| Article/analysis is not finished yet | 6 | 10.9% |
| Low number of participants, therefore underpowered | 6 | 10.9% |
| Discontinued development of the drug | 5 | 9.1% |
| Study was preliminary terminated | 4 | 7.3% |
| Results not statistically significant | 3 | 5.5% |
| Study was presented on conference | 3 | 5.5% |
| Study only intended for development of drug | 3 | 5.5% |
| Results may be published after drug approval | 2 | 3.6% |
| No reason provided/known | 2 | 3.6% |
| Investigators felt not responsible to publish | 2 | 3.6% |
| Journal space restrictions | 1 | 1.8% |
| Sponsor decision | 1 | 1.8% |
| Results were not spectacular | 1 | 1.8% |
| Drug development was transferred to other company | 1 | 1.8% |
| Not included in trial register | 1 | 1.8% |
| Phase 1 study | 1 | 1.8% |
| Validity of data questioned by health authorities | 1 | 1.8% |
| Only reported internally | 1 | 1.8% |
