# Supplementary figures and images for: Non-Publication Is Common among Phase 1, Single-Center, Not Prospectively Registered, or Early Terminated Clinical Drug Trials
Source: PLoS One. 2016 Dec 14;11(12):e0167709. doi: 10.1371/journal.pone.0167709 (PMC5156378; doi:10.1371/journal.pone.0167709)

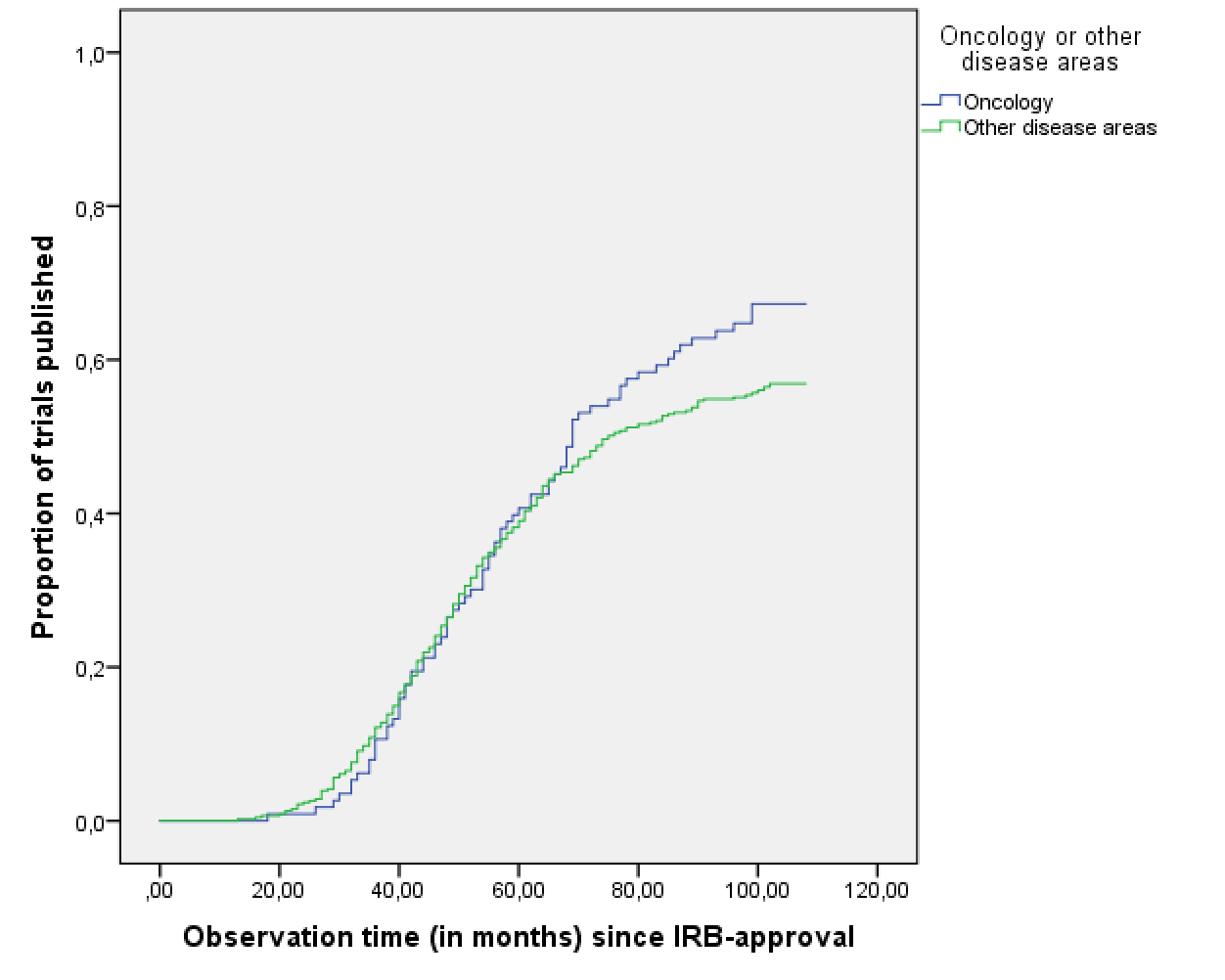

Supplement: S1 Fig — (TIF) [file pone.0167709.s002.tif]

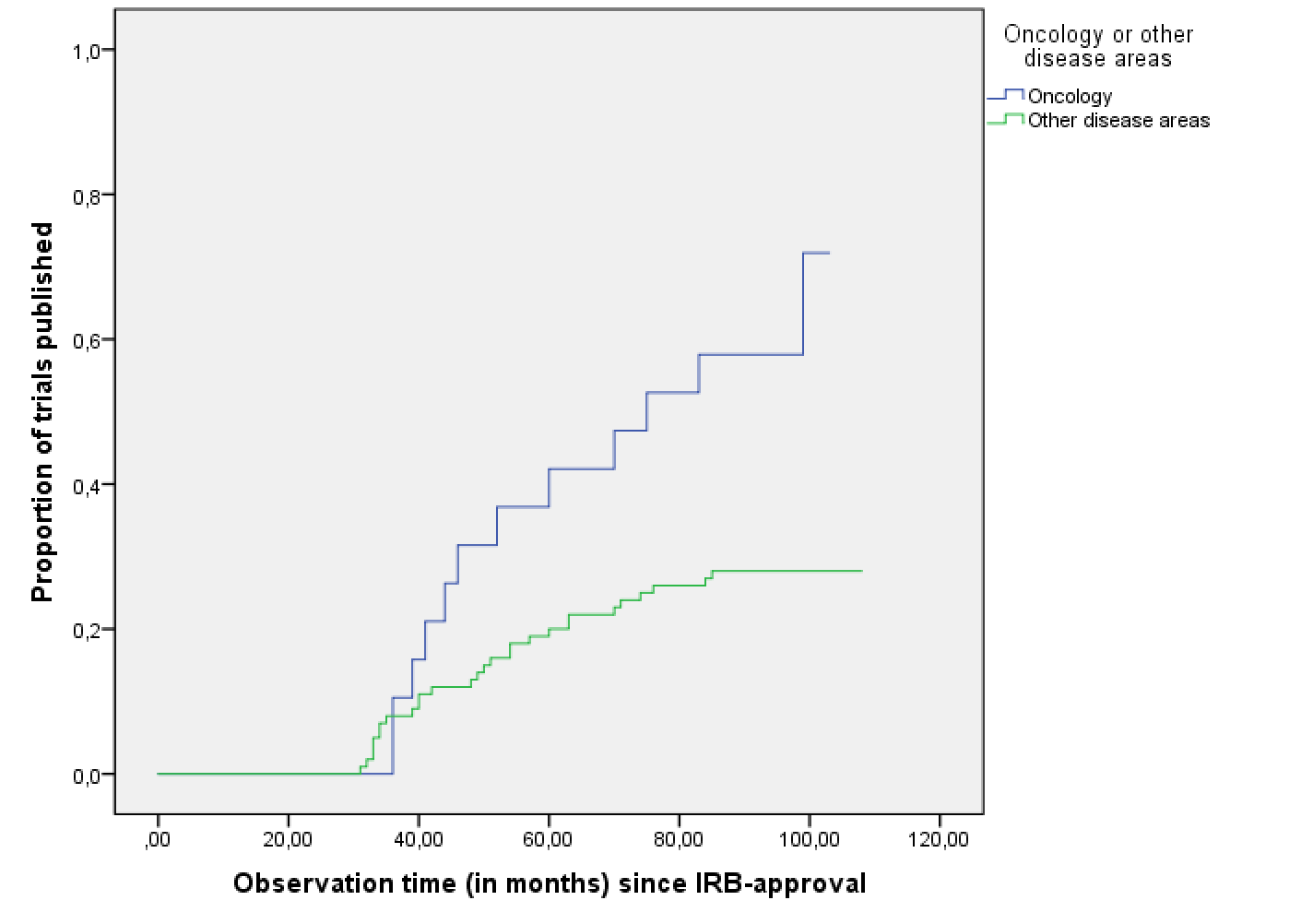

Supplement: S2 Fig — (TIF) [file pone.0167709.s003.tif]

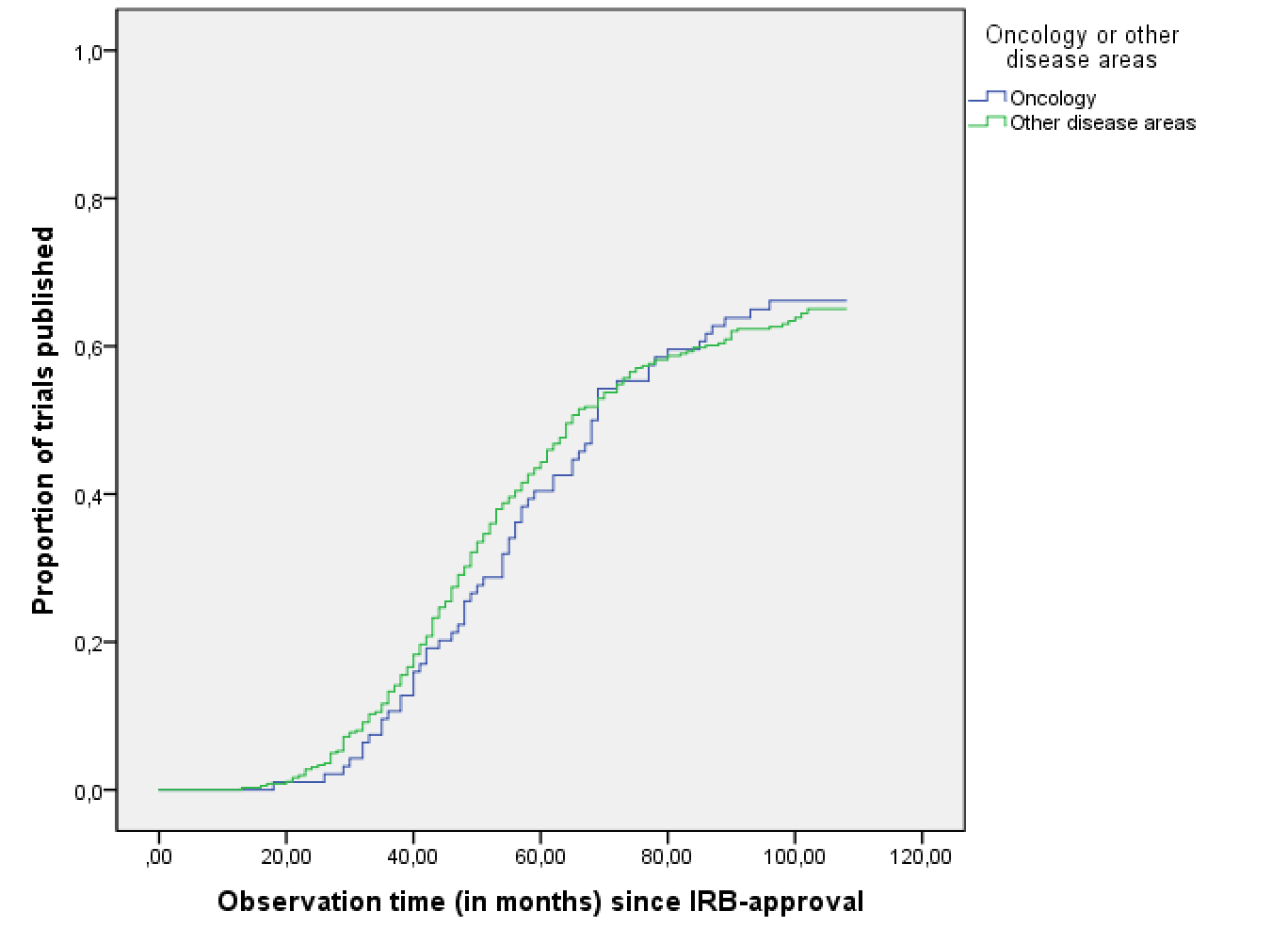

Supplement: S3 Fig — (TIF) [file pone.0167709.s004.tif]
