# Supplementary material for: Non-Publication Is Common among Phase 1, Single-Center, Not Prospectively Registered, or Early Terminated Clinical Drug Trials
Source: PLoS One. 2016 Dec 14;11(12):e0167709. doi: 10.1371/journal.pone.0167709 (PMC5156378; doi:10.1371/journal.pone.0167709)
Supplement: S2 File — (PDF) [file pone.0167709.s006.pdf]

Codebook

Notes

Output Created		24-AUG-2016 15:06:52
Comments		
Input	Data	G:\PhD\CCMO\Study publications\Submission Plos One\Dataset\Data for sharing\SPSS Toetsing online export cohort 2007_for publication_anonymousID.sav
	Active Dataset	DataSet1
	Filter	<none>
	Weight	<none>
	Split File	<none>
	N of Rows in Working Data File	574
Syntax		CODEBOOK Dossier_ID_anonymized [n] Survey_response [n] Prospective_registration [n] Prospective_registration_1year [n] Prospective_registration_0days [n] Registration_atall [n] PhaseofStudyPhase1_dich [n] PhaseofStudy [n] SponsorType [n] CROinvolved [n] DrugCategory_RegularvsOther_dich [n] CentersInvolved [n] TherapeuticEffectExpected_dich [n] TypeofStudy [n] ParticipantCategory_dich [n] ProductRegisteredinNL [n] TherapeuticArea_ICD10 [n] TherapeuticArea_Oncology [n] Prematureending_short [n] Prospective_registration_dich [n] ResultsRegistry [n] Prematureendingofthetrial_complete [n] Outcome_positive_negative [n] EventDate [n] TimeToEvent [s] PublishedasArticle [n] /VARINFO POSITION LABEL TYPE FORMAT MEASURE ROLE VALUELABELS MISSING ATTRIBUTES /OPTIONS VARORDER=VARLIST SORT=ASCENDING MAXCATS=200 /STATISTICS COUNT PERCENT ...
Resources	Processor Time	00:00:00,06
	Elapsed Time	00:00:00,23

Survey_response

		Value	Count	Percent
Standard Attributes	Position	2		
	Label	Response to quesitonnaire		
	Type	Numeric		
	Format	F8		
	Measurement	Nominal		
	Role	Input		
Valid Values	1	Response	181	31,5%
	2	Willing to participate, but unable to track the information	20	3,5%
	3	Refused to participate	6	1,0%
	4	No response	359	62,5%
	5	Requested information sent, but not correct	8	1,4%

Prospective_registration

		Value	Count	Percent
Standard Attributes	Position	3		
	Label	Was the trial prospectively registered in clinicaltrials.gov or ISRCTN?		
	Type	Numeric		
	Format	F8		
	Measurement	Nominal		
	Role	Input		
Valid Values	1	Registered 1 month or more before IRB-approval	215	37,5%
	2	Registered less than 1 month before IRB-approval	106	18,5%
	3	Not registered	253	44,1%

Prospective_registration_1year

		Value	Count	Percent
Standard Attributes	Position	4		
	Label	Registration within 1 year of IRB-approval		
	Type	Numeric		
	Format	F8		
	Measurement	Nominal		
	Role	Input		
Valid Values	1	within one year	297	51,7%
	2	no or after one year	277	48,3%

Prospective_registration_0days

		Value	Count	Percent
Standard Attributes	Position	5		
	Label	Registration at same day of IRB-approval		
	Type	Numeric		
	Format	F8		
	Measurement	Nominal		
	Role	Input		
Valid Values	1	Same day	178	31,0%
	2	Not or later	396	69,0%

Registration_atall

		Value	Count	Percent
Standard Attributes	Position	6		
	Label	Registered or not (regardless timing of registration)		
	Type	Numeric		
	Format	F8		
	Measurement	Nominal		
	Role	Input		
Valid Values	1	Registered	321	55,9%
	2	Not registered	253	44,1%

PhaseofStudyPhase1_dich

		Value	Count	Percent
Standard Attributes	Position	7		
	Label	Phase 1 or other		
	Type	Numeric		
	Format	F8		
	Measurement	Nominal		
	Role	Input		
Valid Values	1	Phase 1	119	20,7%
	2	Other phase	455	79,3%

PhaseofStudy

		Value	Count	Percent
Standard Attributes	Position	8		
	Label	Phase of Study		
	Type	Numeric		
	Format	F8		
	Measurement	Nominal		
	Role	Input		
Valid Values	1	Phase I	119	20,7%
	2	Phase II	130	22,6%
	3	Phase III	172	30,0%
	4	Phase IV	57	9,9%
	5	Other	96	16,7%
	6	Not applicable	0	0,0%

SponsorType

		Value	Count	Percent
Standard Attributes	Position	9		
	Label	Type sponsor		
	Type	Numeric		
	Format	F8		
	Measurement	Nominal		
	Role	Input		
Valid Values	1	Pharmaceutical industry	352	61,3%
	2	Investigator (industry (co-) funded)	71	12,4%
	3	Investigator (no industry funding involved)	151	26,3%

CROinvolved

		Value	Count	Percent
Standard Attributes	Position	10		
	Label	Is a CRO involved in the study?		
	Type	Numeric		
	Format	F8		
	Measurement	Nominal		
	Role	Input		
Valid Values	1	CRO involved	214	37,3%
	2	No CRO involved	360	62,7%

DrugCategory_RegularvsOther_dich

		Value	Count	Percent
Standard Attributes	Position	11		
	Label	Drug in regular or special category		
	Type	Numeric		
	Format	F8		
	Measurement	Nominal		
	Role	Input		
Valid Values	1	Regular medicine (chemical entity or regular large molecule)	549	95,6%
	2	Other product category involved (vaccine; radiopharmaceutic; somatic cell, genetic, RNA, or other therapy)	25	4,4%

CentersInvolved

		Value	Count	Percent
Standard Attributes	Position	12		
	Label	One or more Centers Involved		
	Type	Numeric		
	Format	F8		
	Measurement	Nominal		
	Role	Input		
Valid Values	1	Single center	249	43,4%
	2	Multicenter, only in the Netherlands	54	9,4%
	3	Multicenter, only within the EU	82	14,3%
	4	Multicenter, in the Netherlands and also outside EU	189	32,9%

TherapeuticEffectExpected_dich

		Value	Count	Percent
Standard Attributes	Position	13		
	Label	Therapeutic effect expected		
	Type	Numeric		
	Format	F8		
	Measurement	Nominal		
	Role	Input		
Valid Values	1	Therapeutic effect expected	356	62,0%
	2	No therapeutic effect expected	218	38,0%

TypeofStudy

		Value	Count	Percent
Standard Attributes	Position	14		
	Label	Intervention or (invasive-) observational		
	Type	Numeric		
	Format	F8		
	Measurement	Nominal		
	Role	Input		
Valid Values	1	Intervention	517	90,1%
	2	Invasive observational	45	7,8%
	3	Non-invasive observational	12	2,1%

ParticipantCategory_dich

		Value	Count	Percent
Standard Attributes	Position	15		
	Label	Participant category		
	Type	Numeric		
	Format	F8		
	Measurement	Nominal		
	Role	Input		
Valid Values	1	Adults and mentally capacitated	532	92,7%
	2	Minor and/or mentally incapacitated	42	7,3%

ProductRegisteredinNL

		Value	Count	Percent
Standard Attributes	Position	16		
	Label	Registration status of drugs in study		
	Type	Numeric		
	Format	F8		
	Measurement	Nominal		
	Role	Input		
Valid Values	1	At least 1 unregistered drug in study	306	53,3%
	2	All drugs are registered, at least one used outside indication	147	25,6%
	3	All drugs are registered and used within indication	121	21,1%
	4	No registration status indicated	0	0,0%

TherapeuticArea_ICD10

		Value	Count	Percent
Standard Attributes	Position	17		
	Label	Therapeutic area ICD-10		
	Type	Numeric		
	Format	F8		
	Measurement	Nominal		
	Role	Input		
Valid Values	1	Oncology	113	19,7%
	2	Diseases of the nervous system	72	12,5%

TherapeuticArea_ICD10

	Value	Count	Percent
3	Endocrine, nutritional and metabolic diseases	58	10,1%
4	Diseases of the circulatory system	62	10,8%
5	Mental and behavioral disorders	37	6,4%
6	Certain infectious and parasitic diseases	42	7,3%
7	Diseases of the blood and blood-forming organs and certain disorders involving the immune system	34	5,9%
8	Diseases of the respiratory system	34	5,9%
9	Diseases of the musculoskeletal system and connective tissue	31	5,4%
10	Diseases of the digestive system	25	4,4%
11	Diseases of the genitourinary system	25	4,4%
12	Other therapeutic areas	41	7,1%

TherapeuticArea_Oncology

		Value	Count	Percent
Standard Attributes	Position	18		
	Label	Oncology or other therapeutic area		
	Type	Numeric		
	Format	F8		
	Measurement	Nominal		
	Role	Input		
Valid Values	1	Oncology	113	19,7%
	2	No oncology	461	80,3%

Prematureending_short

		Value	Count	Percent
Standard Attributes	Position	19		
	Label	Prematurely ended		
	Type	Numeric		
	Format	F8		
	Measurement	Nominal		
	Role	Input		
Valid Values	1	Yes	102	17,8%
	2	No	472	82,2%
Missing Values	3	Unknown	0	0,0%

Prospective_registration_dich

		Value	Count	Percent
Standard Attributes	Position	20		
	Label	Trial was prospectively registered		
	Type	Numeric		
	Format	F9		
	Measurement	Nominal		
	Role	Input		
Valid Values	1	Yes	215	37,5%
	2	No	359	62,5%

ResultsRegistry

		Value	Count	Percent
Standard Attributes	Position	21		
	Label	Was a summary of results uploaded in the registry?		
	Type	Numeric		
	Format	F3		
	Measurement	Nominal		
	Role	Input		
Valid Values	1	Yes	136	23,7%
	2	No	438	76,3%

Prematureendingofthetrial_complete

		Value	Count	Percent
Standard Attributes	Position	22		
	Label	Stage of progress		
	Type	String		
	Format	A26		
	Measurement	Nominal		
	Role	Input		
Valid Values	Any follow-up unknown		22	3,8%
	No		360	62,7%
	Started, follow-up unknown		90	15,7%
	Yes		102	17,8%

Outcome_positive_negative

		Value	Count	Percent
Standard Attributes	Position	23		
	Label	Direction of result of the trial		
	Type	Numeric		
	Format	F12		
	Measurement	Nominal		
	Role	Input		
Valid Values	1	Positive	146	25,4%
	2	Negative	65	11,3%
	3	Descriptive/exploratory	146	25,4%
	4	No information	217	37,8%

EventDate

		Value
Standard Attributes	Position	24
	Label	<none>
	Type	Numeric
	Format	EDATE10
	Measurement	Nominal
	Role	Input

TimeToEvent

		Value
Standard Attributes	Position	25
	Label	<none>
	Type	Numeric
	Format	F9.2
	Measurement	Scale
	Role	Input
N	Valid	574
	Missing	0
Central Tendency and Dispersion	Mean	73,0523
	Standard Deviation	27,75555
	Percentile 25	47,0000
	Percentile 50	74,0000
	Percentile 75	100,0000

PublishedasArticle

		Value	Count	Percent
Standard Attributes	Position	26		
	Label	Published as article		
	Type	Numeric		
	Format	F8		
	Measurement	Nominal		
	Role	Input		
Valid Values	0	No	240	41,8%
	1	Yes	334	58,2%
