# Supplementary material for: Non-Publication Is Common among Phase 1, Single-Center, Not Prospectively Registered, or Early Terminated Clinical Drug Trials
Source: PLoS One. 2016 Dec 14;11(12):e0167709. doi: 10.1371/journal.pone.0167709 (PMC5156378; doi:10.1371/journal.pone.0167709)
Supplement: S3 File — Based on our initial search, we sent 4 different questionnaires, depending on whether or not we found that the trial was published, and depending on whether or not we had information on the end of trial (completed as planned or terminated early). (ZIP) [file pone.0167709.s007.zip › Survey4_ENGLISH.pdf]

Questionnaire of research project 'Better Use of Files'

This is the online questionnaire of the research project 'Better Use of Files, of which you were informed by email

There are 9 questions in this survey

Trial identification

1 [1]

What is the NL-number of the trial?

Please fill out the NL-number exactly as it is noted in the email, including the 2 dots. Example: NL12345.678.90

Investigators who received emails for more than one trial, please complete a separate survey for each trial *

Please check the format of your answer.

Please write your answer here:

2 [8]What was the date that inclusion started? *

Please write your answer(s) here:

In the Netherlands:

In case of an international multicenter trial: for the whole trial:

3 [4]Has the trial prospectively been registered at a public register, e.g. www.clinicaltrials.gov or ISRCTN? By prospectively we mean before recruitment of the first participant. *

Please choose **only one** of the following:

- Yes

○ No

4 [6]Note the trial identification number for the register at issue (e.g. NCT12345678) *

Only answer this question if the following conditions are met:
Answer was 'Yes' at question '3 [4]' (Has the trial prospectively been registered at a public register, e.g. www.clinicaltrials.gov or ISRCTN? By prospectively we mean before recruitment of the first participant.)

Please write your answer here:

Publication of the trial

The final set of questions addresses the publication of the trial. By publication we mean publishing the methods and results of the trial in a peer-reviewed journal

5 [1]We may have identified a publication of the trial. Is the trial indeed published as peer-reviewed article? *

Please choose **only one** of the following:

○ Yes

○ No

6 [2]

Please note the title(s), journal(s), journal edition(s), and publication date(s) of the article(s) *

Only answer this question if the following conditions are met:
Answer was 'Yes' at question '5 [1]' (We may have identified a publication of the trial. Is the trial indeed published as peer-reviewed article?)

Please write your answer here:

7 [3]

Why has the trial not been published? *

Only answer this question if the following conditions are met:
Answer was 'No' at question '5 [1]' (We may have identified a publication of the trial. Is the trial indeed published as peer-reviewed article?)

Please choose all that apply and provide a comment:

- ☐ Other priorities
- ☐ Resultaten niet statistisch significant Results not statistically significant
- ☐ Resultaten niet klinisch relevant Results not clinically relevant
- ☐ Manuscript is afgewezen door tijdschrift(en) Manuscript was rejected by journal(s)
- ☐ Other reasons (please specify):

8 [4]

Are the results of the trial reported in a different way than in a peer reviewed journal?

Please choose **only one** of the following:

- Yes (Please specify where, e.g. results reported on clinicaltrials.gov + registration number)
- No

Make a comment on your choice here:

Einde vragenlijst

9 [3]Thank you for completing the questionnaire. You can add any additional comments here. Please do not forget to click on the button 'Submit' at the bottom of this page.

Please write your answer here:

Many thanks for your collaboration! Your answers have been saved. Please do not fill out the questionnaire again.

In case of unforeseen errors or system malfunction, please contact Sander van den Bogert: s.van.den.bogert@ccmo.nl

Submit your survey.
Thank you for completing this survey.
